# Genome report of *Peribacillus simplex* strain IMGN9 isolated from soil

**DOI:** 10.1128/mra.00860-24

**Published:** 2024-11-26

**Authors:** Hyeonsik Yoon, Jaeyeon Lee, Yeongjun Kim, Jeong A. Han, Ho-Seok Lee, Eun Yu Kim

**Affiliations:** 1 Center for Genome Engineering, Institute for Basic Science, Daejeon, South Korea; 2 Department of Biology, College of Science, Kyung Hee University, Seoul, South Korea; 3 Gyeonggido Agricultural Research and Extension Services, Hwaseong, South Korea; 4 Division of Natural and Applied Sciences, Duke Kunshan University, Kunshan, China; 5 Environment Research Center, Duke Kunshan University, Kunshan, Jiangsu, China; University of Maryland School of Medicine, Baltimore, Maryland, USA

**Keywords:** *Peribacillus simplex*, biocontrol, PGPR, bioremediation

## Abstract

Here, we report the draft genome sequence of *Peribacillus simplex* IMGN9, a bacterium renowned for its biocontrol activity, plant-growth-promoting properties, and bioremediation potential. This study aims at the potential utilization of the bacterium and its mechanisms, thus attempting to improve our understanding of biocontrol and bioremediation.

## ANNOUNCEMENT

*Peribacillus simplex*, a gram-positive, endospore-forming, rod-shaped, aerobic bacterium, exhibits diverse habitats. Prior to its reclassification in 2020, *P. simplex*, the type species of the genus *Peribacillus*, was known as *Bacillus simplex* (1). *P. simplex* possesses distinctive biological properties, including plant-growth-promoting, biocontrol, and bioremediation capabilities. Its biocontrol activities against fungi, nematodes, and bacteria effectively defend plants against phytopathogens. Furthermore, treatment with *P. simplex* can induce systemic resistance through diverse mechanisms (2). In the field of bioremediation, the bacterium can reduce contaminants including multiple heavy metals (3) and low molecular weight polyaromatic hydrocarbons (4). In conclusion, this genome announcement for IMGN9 could improve our understanding of the genetic basis of this versatile bacterium.

IMGN9 was isolated from soil collected from Jipyeong-myeon, Yangpyeong-gun, Gyeonggi-do, Republic of Korea (37°47’34.1”N, 127°62’80.9”E) in 2019. The soil sample was diluted 1:10 and then further diluted 1:1,000 in distilled water. The diluted sample was spread onto a tryptic soy agar (TSA) plate and incubated at 15°C for 16 hours in the dark. The culture was then streaked twice onto fresh TSA plates, following the same protocol, to obtain a pure colony.

Genomic DNA was extracted from the tryptic soy broth culture using the MagAttract HMW DNA Kit (QIAGEN, Cat No. 67563) and quantified with a Qubit 2.0 fluorometer. To generate libraries, 5 µg of genomic DNA was sheared using the Megaruptor 3 (Diagenode) according to the protocols provided by the manufacturer, and small fragments were removed by using AMPure XP bead purification. The SMRTbell library was constructed using SMRTbell Express Template Preparation Kit 2.0 (PacBio, Cat No. 100–938-900) according to the manufacturer’s instruction and sequenced using the Sequel I (Pacific Biosciences) sequencing platform. To improve the quality of the genome, automated systems within the PacBio SMRT Link platform were used for read quality control, error correction, and adapter trimming.

The PacBio sequencing of IMGN9 generated 295,083 reads with a mean length of 6,788 base pairs and an N50 value of 8,175 base pairs. Genomic contamination was assessed using the ContEst16S (5) algorithm, with no contamination detected. Default parameters were applied unless otherwise specified. The HiFi reads were assembled *de novo* using SMRT Link 10.1.0 with the Microbial Assembly protocol, yielding three linear contigs totaling 5,679,363 base pairs with a coverage of 308.58×. This included one draft genome contig of 5,656,011 base pairs and two putative chromosome fragments of 13,751 and 9,601 base pairs, respectively (6).

The annotation with the NCBI Prokaryotic Genome Annotation Pipeline v6.7. predicted 5,240 protein-coding genes, 100 tRNA genes, and 46 rRNA genes. For more functional annotation, predicted coding proteins were compared against Protein Family Models (7, 8). The draft genome of IMGN9 comprises 5,679,363 base pairs, with an average GC content of 40.36% and an estimated completeness of 99.1%. It includes three contigs: 5,656,011 base pairs with a GC content of 40.34%, 13,751 base pairs with a GC content of 43.33%, and 9,601 base pairs with a GC content of 47.04%. The N50 value of the draft genome was 5,656,011 base pairs.

As the world grapples with contamination from agricultural chemicals and other pollutants, the pressing need for sustainable alternatives and purification methods has surged. Biocontrol and bioremediation offer promising solutions to address these challenges by utilizing living organisms to control pathogens and reduce the concentration of contaminants(9–14). IMGN9 has the potential to be implicated in the control of phytopathogens and the bioremediation of heavy metals and low molecular weight polyaromatic hydrocarbons, such as fluorene and phenanthrene (2), which can pose risks to human health.

## Data Availability

This Whole Genome Shotgun project has been deposited at DDBJ/ENA/GenBank under the accession JBEVOZ000000000. The version described in this paper is version JBEVOZ010000000. The BioProject database accession number is PRJNA1114006, and the BioSample accession number is SAMN41474202. The Sequence Read Archive (SRA) information is available under the accession number SRX24856517 and SRX24856518.
